# Self-reported morbidities, nutritional characteristics, and associated factors in institutionalized and non-institutionalized older adults

**DOI:** 10.1186/s12877-021-02067-3

**Published:** 2021-02-24

**Authors:** Abdelhafid Benksim, Rachid Ait Addi, Elhassania Khalloufi, Aziz Habibi, Mohamed Cherkaoui

**Affiliations:** 1 Nursing Care Department, Higher Institute of Nursing Professions and Health Techniques (ISPITS-M), Health department of Marrakech-Safi region, 40000 Marrakech, Morocco; 2 Laboratory of Human Ecology, Faculty of sciences Semlalia, Cadi Ayyad University, 40000 Marrakesh, Morocco

**Keywords:** Self-reported morbidity, Older adults, Institutionalization, Nutritional status, Morocco

## Abstract

**Background:**

As the world’s population ages and people live longer, it seems important to ensure that older people have a good quality of life and positive subjective well-being. The objective of this study is to determine socio-economic, health and nutritional characteristics of institutionalized and non-institutionalized elders in the province of Marrakech.

**Methods:**

This study was conducted among 368 older adults in the province of Marrakech between March 2017 and June 2019. Of all participants, 180 older adults reside in a public institution and 188 of them live in their own homes. Data on health conditions, nutritional status, functional and socio-economic characteristics were collected. Data was analyzed using SPSS Statistics for Windows, Version 16.0. Statistical significance was set at *p* < 0.05.

**Results:**

Institutionalized elders were illiterate (80.0%), had low incomes (95.5%), and unmarried (73.3%), they reported also no children (56.1%) and no health insurance (98.9%). Institutional residents suffered from malnutrition (22.2%), hearing impairments (35.6%) and severe edentulism (43.3%). There was no significant difference between both groups on daily activities and depression. A multivariate analysis identified a model with three significant variables associated with non-institutionalized elders: health insurance (*P* = 0.001; OR = 107.49), number of children (*P* = 0.001; OR = 1.74) and nutritional status (*p* = 0.001; OR = 3.853).

**Conclusions:**

This study shows that the institutionalization of older adults is considerably induced by various factors such as nutritional problems, lack of health insurance and family structure. To mitigate the effects of this phenomenon, home care strategies and preventive actions should be implemented to delay the institutionalization of older adults and therefore keep them socially active in their own homes.

## Background

Throughout the world, elderly population is growing rapidly. It is predicted to reach 2 billion persons by 2050, raising serious concerns for the planning and management of health systems [1, 2]. Furthermore, the global interest in successful aging is now focused on how to keep people incessantly at home or in the community. However, when the need for care is great, admission to an institution may be unavoidable and necessary. Currently, exceptional changes are occurring around the world as fertility and mortality rates decline and the population ages. An increase in the proportion of older adults is expected in most countries [3]. It affects individuals and communities as they seek to address issues related to health care, social security, caregiving and the burden of diseases and disabilities. This transition, often associated with multiple chronic morbidities, will result in a large increase in the number of older adults requiring adequate nursing care [4].

However, demographic transition is causing dramatic changes in the health profile of older adults who often suffer from multiple illnesses and severe disabilities. This results in high levels of vulnerability and therefore an increased demand for caregivers, as family members are now less available than in the past to do this work [5]. Several Studies have suggested that older people tend to suffer from a large number of psychological, functional and health-related illnesses due to environmental living conditions [6].

Today, the institutionalization of older adults is seen as a burden on all communities, due to high demand, low capacity and increased lack of funds [7]. Therefore, most elders prefer to live at home because they are able to maintain the integrity of their social network, preserve environmental benchmarks and enjoy a better quality of life. Besides, admission to a nursing home is costly in terms of public and private finances [7]. Nonetheless, institutionalization is associated with several negative outcomes such as increased mortality, reduced quality of life and questionable care. The unfavourable results accompanying institutionalization confirmed efforts to prevent or delay placement older adults in nursing homes [8].

Some studies have shown the differences between institutionalized and non-institutionalized elders. Although both groups tend to associate old age with chronic diseases and disabilities, institutionalized elders seem to experience the aging process in a bad way and become increasingly socially and emotionally isolated [9]. However, for non-institutionalized elders, old age seems to be related with activity, freedom, autonomy and the socialization phase as a time to make new friends [9].

Previous studies have shown that the main predictors of institutionalization are being female, being single, being illiterate, without family support, and having cognitive or physical impairment [4, 10]. Thus, institutionalized older adults are more likely to have low socioeconomic status, to be widowed or single, uninsured, malnourished, and often suffer from chronic illnesses that make them more dependent in their daily lives [4, 10].

In Morocco, older adults are expected to represent 23.2% in 2050 against 9.4% in 2014 [11]. The aging urges the government to develop new strategies to meet the needs of specialized care of older adults. Thus, there is a significant lack of specialized care intended for this category because there were few residences entirely dedicated throughout the country, managed by non-profit associations [11]. Furthermore, many older adults are now living alone due to massive youth immigration, falling death rates, major advances in healthcare and social change. It is expected that an increased demand for nursing homes and subsidized care will be necessary in the future. Our main objective in this study was to determine the differences between institutionalized and non-institutionalized older adults and infer the self-reported morbidity, nutritional status and associated factors in the province of Marrakech.

## Methods

### Ethic statement

A group of 368 older persons aged 60 years and over were selected by a non-probability sampling between March 2017 and June 2019 through a face-to-face interview. Study protocol was explained and informed consent was obtained. The Illiterate participants were accompanied by a third party to explain themselves (brother, sister, caregiver, or a member of the family). In this case, the consent was signed by both people. We also proceeded to obtain consent in Arabic dialect to facilitate understanding. The protocol study was approved by the ethics committee of health authorities in Marrakech’s province.

### Data collection

The target population was older adults living in their own homes and those residing in public institution. The exclusion criteria were persons who suffered from severe dementia, and neuropsychological disorders. Inclusion criteria were the individuals aged ≥60 years. Collection tool was tested with a pilot group as close as possible to the target population. All data were collected by trained nursing students.

As we do not have detailed information on the actual proportion of older adults living in the province of Marrakech (p is unknown), we use *p* = 0.50 to estimate the size of our final sample (384 are needed). However, after excluding some people who did not meet the inclusion criteria, our final sample was set at 368 participants.

Furthermore, a group of 368 participants was selected by a simple random sampling method (including 180 older adults in public institution (Dar-lbir-waihssan) and 188 are living at home (alone or with family members). This public institution is the resort for older adults and homelessness. It functions as a public non-profit institution provides such services as accommodation, meals, full-time nursing coverage, health care and rehabilitation.

### Variables and modalities

The data is collected through an interview guide, which includes various information such as age, sex, origin (urban or rural), number of children, marital status, and socioeconomic status (SES), which included education, previous occupation and health insurance (covered or not). Education status was coded into four categories: 1) Illiterates: those who never went to school, 2) Primary level: those who attended the primary-school; 3) Secondary level: those who have enrolled in middle school and/or high school without getting the baccalaureate certificate; 4) Tertiary level: those who went to university. Economic levels are estimated on the basis of monthly income relating to previous occupations: 1) Category with an income below 3500 dirhams per month (MAD): secretary, cashier, janitor, server, housekeepers and maids, etc.. 2) Category with middle income ranging between 3500 and 5308 MAD: Police officer, taxi driver, teachers, nurses, etc.. 3) Category with income exceeding 5308 MAD: Financial manager, engineer, professor, physician, lawyer, accountant, etc.

Physical disabilities were assessed when a person performed basic tasks of daily living (ADLs). There are six basic activities including bathing, dressing, feeding, transferring, continence and toileting. Hence, three categories are made: 1) no difficulties doing everything, 2) slight difficulty to do some things, 3) difficulty doing everything. We used mini nutritional assessment–Short Form (MNA-SF) to measure nutritional status (ranging from 0 to 14 points). MNA-SF scale is interpreted as follows: 1) Malnutrition: 0 to 7points, 2) Risk of malnutrition: 8 to 11 points and 3) Normal: 12 points or greater. Depressive symptoms were assessed with 15-items version of Geriatric Depression Scale (GDS-SF), ranging from 0 to 15 points: 1) Normal: 0–5, 2) Moderate depression: 6–10 and 3) Severe depression. Participant’s health status was assessed on the basis of an anamnesis, or a clinician’s evaluation, and/or through a brochure providing information (type and number of illnesses). Edentulism was estimated by the question “How many missing teeth do you have?”. Three categories were categorized: 1) Good status: relatively no problem of dentition, 2) Partial edentulism: more than half of teeth are deteriorated and 3) Severe edentulism: all teeth are missed or must be removed.

### Statistical analyses

The data were examined using SPSS version 16.0 (Inc., Chicago, IL, USA). A one-sample Kolmogorov-Smirnov test was used to analyse normality for continuous variables. Chi Square test was used to study relationships between categorical variables. Student’s t-test was used to compare means between both groups. Multiple linear regressions identified the independent factors that affect institutionalized elders. Odds ratio (OR) 95%CI was used to show the strength of relationship between independent variables. Wald χ2 statistic is used to test the significance of individual coefficients for verifying the true values. Statistical significance was defined as *p* < 0.05.

## Results

### Socio-demographic characteristics

A total of 368 older adults were included in this study, 188 are living in their own homes and 180 in a public establishment in the province of Marrakech. Table 1 summarizes the socio-demographic and economic characteristics. The mean age of institutionalized and non-institutionalized participants was 69.19 years (SD = 9.12) and 70.42 years (SD = 8.94), respectively. Of all the respondents, 45.9% were male and 54.1% were female, with the majority reporting low socio-economic status and poor health. In comparison to non-institutionalized, institutionalized elders reported being illiterate (80.0%), unemployed or low-income (95.5%), unmarried (73.3%), without health insurance (98.9%), and more than 50% of them had no children.

**Table 1:** Socio-economic and demographic characteristics of institutionalized and non-institutionalized elders

	Institutionalized elders. *n* = 180	Non-institutionalized elders *n* = 188	*P-value*
Age (years ± SD)	69.19 ± 9.12	70.42 ± 8.94	0.748
Sex n (%)			0.364
Male	87 (48.3)	82 (43.6)	
Female	93 (51.7)	106 (56.4)	
Marital status. n (%)			0.001
Without partner	132 (73.3)	72 (38.3)	
With partner	48 (26.7)	116 (61.7)	
Education status. n (%)			0.001
Illiterate	144 (80.0)	128 (68.1)	
Primary	23 (12.7)	20 (10.6)	
Secondary	9 (5.1)	5 (2.6)	
Tertiary level	4 (2.2)	35 (18.6)	
Health insurance. n (%)			0.001
None	178 (98.9)	113 (60.1)	
Yes	2 (1.1)	75 (39.9)	
Previous income per month. n (%)			0.001
With low income	172 (95.5)	146 (77.6)	
With middle income	6 (3.3)	13 (6.9)	
With high-income	2 (1.1)	29 (15.4)	
Origin. n (%)			0.176
Urban	84 (46.7)	101 (53.7)	
Rural	96 (53.3)	87 (46.3)	
Number of children. n (%)			0.001
1 or more	79 (43.9)	181 (96.3)	
0	101 (56.1)	7 (3.7)	

Abbreviations: *SD* Standard deviation; (): absolute frequency

### Health characteristics and nutritional status

Table 2 presents clinical, nutritional characteristics and self-reported morbidities of participants. According to the MNA-SF, people living in institutions are three times more likely to be malnourished than those who are not, with an average score of 9.42+/− 1.93. Moreover, more than half of participants suffered from at least two chronic diseases, especially those who were in institutions, with no significant differences between both groups (*P* > 0.05). Thus, non-institutionalized elders were more likely to suffer from hypertension, heart diseases (37.8%), musculoskeletal diseases (33.5%), gastrointestinal diseases (20.7%), compared to institutionalized elders, who suffered also from visual disorders (32.8%) and metabolic disorders (22.2%) with no significant differences between both groups. However, institutionalized subjects were significantly more likely to develop severe toothlessness (43.3%) and hearing impairment (35.6%) (*P* < 0.05). Self-reported activities disclose that 12.8% of institutionalized had difficulty to perform their daily tasks without any assistance compared to those living at home (*p* = 0.385). Therefore, when analysing GDS-SF, a proportion of severe depression is slightly assessed in institutionalized elders than those living at home (*P* = 0.313). Table 3 provides a design of independent variables significantly associated with institutionalization. This multivariate analysis divulges that health insurance (*P* = 0.001; OR = 107.49; 95%CI: 14,292–808.524), number of children (*P* = 0.001; OR = 1.74; 95%CI: 1.498–2.023) and nutritional status (*P* = 0.001; OR = 3.853; 95%CI: 2.152–6.898) are relatively the main predictive variables associated with institutionalized elders.

**Table 2:** Clinical characteristics and frequency of self-reported morbidity among institutionalized and non-institutionalized older adults

	Institutionalized elders *n* = 180	Non-institutionalized elders *n* = 188	*P*-value
MNA-SF score	9.42 ± 1.93	12.55 ± 1.42	0.016
MNA-SF. n (%)			0.001
Malnutrition: 0 to 7 points	40 (22,2)	15 (8,0)	
Risk of malnutrition: 8 to 11 points	114 (63,3)	66 (35,1)	
Normal: 12 points or greater	26 (14.4)	107 (56.9)	
Mean of self-reported morbidity	1.63 ± 0.89	1.66 ± 1.05	0.142
Number of self-reported morbidity			0.218
None	8 (6.2)	16 (11.5)	
1	38 (29.5)	45 (32.4)	
2 or more	83 (64.3)	78 (56.1)	
Morbidities Perceived. n (%)			
Heart diseases/hypertension (I10-I15)	53 (29.4)	71 (37.8)	0.091
Respiratory diseases (J40-J47)	7 (3.9)	13 (6.9)	0.201
Infectious diseases (B95-B98)	6 (3.3)	1 (0.5)	0.049
Skin diseases (B35-B49)	5 (2.8)	3 (1.6)	0.437
Musculoskeletal diseases (M00-M99)	47 (26.1)	63 (33.5)	0.121
Gastrointestinal diseases (K00 - K93)	28 (15.6)	39 (20.7)	0.197
Metabolic disorders (E00 - E90)	40 (22.2)	38 (20.2)	0.637
Kidney diseases (N00 - N99)	17 (9.4)	11 (5.9)	0.194
Visual disorders (H00 - H59)	59 (32.8)	60 (31.9)	0.860
Degree of dehydration. n (%)			0.916
Severe dehydration	42 (23.3)	43 (22.9)	
Moderate dehydration	138 (76.7)	145 (77.1)	
Hearing status. n (%)			0.014
Without problem	116 (64.4)	132 (70.2)	
Hearing impairments (H90-H95)	64 (35.6)	56 (29.8)	
Dental status (K00-K14). n (%)			0.043
Good status	19 (10.6)	32 (17.0)	
Partial edentulism	83 (46.1)	95 (50.5)	
Severe edentulism	78 (43.3)	61 (32.5)	
Activity of daily living (ADLs). n (%)			0.385
No difficulties in everything	113 (62.8)	106 (56.4)	
A moderate difficulties	44 (24.4)	50 (26.6)	
Difficulties in everything	23 (12.8)	32 (17.0)	
GDS-SF score	5.64 ± 4,13	5.01 ± 3,87	0.513
GDS (Short form). n (%)			0.313
Normal: 0–5	106 (58.9)	115 (61.2)	
Moderate depression: 6–10	41 (22.8)	49 (26.1)	
Severe depression: 11–15	33 (18.3)	24 (12.8)	

Abbreviations: *GDS* Geriatric Depression Scale, *MNA-SF* Mini nutritional assessment short form test; (): absolute frequency, (−): code of CID-10 to transpose diagnoses of diseases and related health problems

**Table 3:** Variables independently associated with institutionalized (*n* = 180) and non-institutionalized older adults (*n* = 188) according to the multiple logistic regression model

	β	Wald	P-value	OR (95% CI)
Origin: (Urban)	0.197	0.288	0.591	1.218 (0.593–2.501)
Marital status: (Without partner)	0.512	1.756	0.185	1.669 (0.782–3.558)
Education status: (Illiterate)	0.252	0.289	0.591	1.286 (0.514–3.218)
Health insurance: (None)	4.677	20.643	0.000	107.495 (14.292–808.524)
Previous occupation level: (low income)	−0.645	2.670	0.102	0.525 (0.242–1.137)
Number of children: (None)	0.554	52.407	0.000	1.741 (1.498–2.023)
Heart diseases/hypertension (I10-I15)	−0.616	2.465	0.116	0.540 (0.250–1.165)
Infectious diseases (B95-B98)	−3.581	5.063	0.240	0.028 (0.001–0.630)
Musculoskeletal diseases (M00-M99)	0.586	2.364	0.124	1.796 (0.851–3.790)
Gastrointestinal diseases (K00 - K93)	0.133	0.085	0.770	1.142 (0.469–2.780)
Hearing status/ Hearing impairments (H90-H95)	0.188	0.337	0.561	1.206 (0.641–2.272)
Dental status/ severe edentulism (K00-K14)	−0.061	0.121	0.728	0.941 (0.667–1.327)
MNA-SF: (malnutrition) (E40-E46)	1.349	20.597	0.000	3.853 (2.152–6.898)

Abbreviations: *MNA-SF* Mini nutritional assessment short form, *β* Coefficient, *P* Significance level of the Wald test, *OR* Odds ratio and *CI* Confidence interval; (−): code of ICD-10 to transpose diagnoses of diseases and related health problems

## Discussion

To our knowledge, few studies have been devoted to older adults in Morocco. This cross-sectional study explores the socio-economic, demographic and health status of older adults in the province of Marrakech.

### Socio-demographic and economic characteristics

In terms of socio-economic status, older adults living in institutions are less educated and more likely to have lower occupational status, to be single or widowed, to be uninsured and dependent on their children and relatives than non-institutionalized elders. These findings have been corroborated with some studies elsewhere [2–6]. Old age and being single have been shown to be significantly associated with an increased risk of institutionalization [2, 4, 10]. Furthermore, when comparing groups of institutionalized elders, the average age does not exceed 71 years; this result does not corroborate with those elsewhere [12, 13]. In some studies, the risk of institutionalization was higher in persons over 80 years old [2, 11, 12]. Analogous to our results, a high proportion of older adults living alone or unmarried are more likely to be institutionalized [2.14]. In addition, absence of children was relevant in institutionalization process, as older adults may have difficulties in daily life to receive care at home due to changes in family profile (Absent or emigrated children). Some findings show that the decision at an advanced age to remain single, widowed or divorced increases the risk of institutionalization [2, 4, 10, 12]. Furthermore, older adults prefer to live at home because they are able to maintain integrity of their social network, keep their bearings and enjoy a better quality of life [2, 12]. It was found that aging in place and family care are seen as ideal approaches to long-term care because they guarantee the values of dignity, autonomy, family integrity and social continuity [13, 14]. Therefore, the negative outcomes of institutionalization may support efforts to prevent or delay their placement in nursing homes. Additionally, uninsured elders are more likely to move to facilities that provide full-time nursing care. A similar study has shown that the occupancy rate of care services by the uninsured older adults was lower than those insured [14]. Studies have shown that the majority of people living in institutions are uninsured in the Middle East [2, 12, 15].

### Health characteristics and nutritional status

Many chronic diseases and disabilities are initially observed in institutionalized older adults. However, multivariate analysis showed that the majority of independent variables lost their significance. Previous studies indicate that multiple morbidities are the common burden of institutionalization [2, 16]. Another studies indicate that the decision to move to facilities is often made in cases of severe cognitive or functional impairments, due to a lack of nursing care and family support at home [16]. Furthermore, based on MNA-SF scores, institutionalized elders were at risk of malnutrition, while 14.4% were malnourished. This prevalence of malnutrition varies from one population to another. It is often the result of unhealthy diet or nutrient deficiencies as well as unintentional neglect by caregivers. In Sweden, one third of older adults living in nursing homes suffer from malnutrition [17]. In some study, older adults living in long-term care homes are nutritionally vulnerable, often consuming insufficient energy, macro and micronutrients to maintain their health and function [18].

As a vicious circle, inadequate nutrition not only contribute to progression of existing chronic diseases such as osteoporosis or mental disorders, but can also predispose individuals to various acute health problems such as infection or dehydration [17].

Furthermore, institutionalization imposes changes in the daily life of older adults, including eating habits and frailty, due to reduced acceptance of food, resulting in the compromise of their nutritional status [17]. For this reason, one of the most important requirements of care is to provide an adequate diet that includes all essential nutrients. Some studies found that majority of institutional residents were at risk of malnutrition and 19.4% were malnourished [19]. Thus, a similar study found that the prevalence of malnutrition was higher among women and institutional elders [20].

In the same context as our study, residents of institutions are exposed to an increased risk of malnutrition for a variety of reasons, including sensory disturbances, chewing and swallowing problems, mobility restrictions, cognitive and chronic diseases requiring various treatments [21]. Furthermore, some changes in body composition, organ function, adequate energy intake and ability to access food are associated with aging, may contribute to malnutrition [22]. Hence, studies have shown that malnutrition in older adults can result from disorders of the gastrointestinal and endocrine systems, loss of taste and smell and inadequate nutrition [22]. In addition, the poor dental health or chewing and swallowing problems may exacerbate the malnutrition in institutionalized elders [22]. Based on some studies, the risk of malnutrition has shown a positive correlation with the number of existing geriatric syndromes. Thus, depression, dementia, functional dependence and multiple co-morbidities were associated with poor nutritional status [22].

In our study, most of older adults suffered from chronic illnesses, functional disorders and depressive symptoms with no significant difference between both groups. Studies published elsewhere have reported the same results [23, 24]. These conditions will result in an increased need for long-term care and support services for older adults as active members of their society.

When we analysed oral health, a significant rate of edentulism was observed in institutional residents. Studies have shown similar findings in Spanish, Indonesian and Brazilian older adults [23, 25, 26]. Hence, these findings are often due to unhealthy behaviours and attitudes of Moroccans people toward oral hygiene. In addition, illiteracy, poverty, lack of health insurance, poor hygiene, malnutrition, diabetes and hypertension increase the frequency of oral diseases in institutions [23, 25–27].

Regarding depression, the majority of institutional elders suffered from severe depression. Similar results have been reported in some studies which included older adults living in different nursing homes [2, 28]. The high prevalence of depression in institutions could be reduced by improving their nutritional status, their belief in a just world, their quality of life and their social relationships with friends and relatives [2].

In this investigation, there are many limitations due to the fact that the assessment of health status was based on the perception of older adults, including dehydration, hearing and oral condition. In addition, sampling is carried out on small groups.

## Conclusion

The study at hand shows the differences between institutionalized and non-institutionalized elders. Besides, most participants have lower socioeconomic and poorer health status, functional disabilities and some depressive symptoms. However, elders in institutions are more likely to suffer from poor nutritional status, mouth and hearing problems. These findings encourage health care providers and government agencies to enhance the quality of life, with supplementary attention giving to older women and those living in institutions.

## Data Availability

The data will be made available on reasonable request to the corresponding author.

## Abbreviations

GDS: Geriatric Depression Scale
MNA-SF: Mini nutritional assessment short form test
ICD-10: International Statistical Classification of Diseases
ADLs: Activity of daily living
